# The first causal inference analysis of the Catalan Arthroplasty Register shows a positive effect of antibiotic‐loaded bone cement on knee prosthesis survival

**DOI:** 10.1002/jeo2.70574

**Published:** 2025-12-17

**Authors:** Borja Velasco‐Regulez, Sergi Gil‐Gonzalez, Jesus Cerquides

**Affiliations:** ^1^ Data and AI Area Agency for Health Quality and Assessment of Catalonia (AQuAS) Barcelona Spain; ^2^ Learning Systems Artificial Intelligence Research Institute (IIIA‐CSIC) Bellaterra Spain; ^3^ Institut d'Investigació i Innovació Parc Taulí (I3PT) Sabadell Spain; ^4^ Universitat Autònoma de Barcelona Sabadell Spain

**Keywords:** antibiotic‐loaded bone cement, causal inference, causal survival analysis, total knee arthroplasty

## Abstract

**Purpose:**

The survival of a knee prosthesis is one of the most important indicators of the success or failure of a knee arthroplasty. An intervention that could increase prosthetic survival is the use of antibiotic‐loaded bone cement (ALBC) during primary surgery, but the evidence for this is not conclusive. The question of whether such an intervention increases prosthetic survival is a causal one, and yet it has never been addressed with causal methods in the observational studies literature. This constitutes a serious limitation, as there is growing evidence that the best‐suited framework for addressing causal questions with observational data is causal inference.

**Methods:**

In the present study, causal inference methods were employed to answer the research question of whether ALBC increases prosthetic survival. In particular, directed acyclic graphs were used for identification and causal survival forests were used to estimate the effect of interest. The rationale behind these methods is provided in the main text, and technical details are provided in Supporting Information: File S3. Data from the Catalan Arthroplasty Register were analysed.

**Results:**

ALBC had an effect of increasing the overall prosthetic survival by 8% after 120 months of follow‐up. The intervention had a positive effect across all the subgroups of the population defined by confounding variables, but the effect was greater in men, young patients, patients with rheumatoid arthritis or obesity, and patients who smoked or abused alcohol. The chosen causal assumptions had an impact on the obtained results, empirically showing the importance of using a causal framework.

**Conclusions:**

ALBC increased knee prosthesis survival among patients in the Catalan public healthcare system. Causal inference methods are the most appropriate for answering causal questions about the effect of ALBC on prosthetic survival when the analysed data are observational.

**Level of Evidence:**

Level III.

**Abbreviations**
ALBC: antibiotic‐loaded bone cement
ATE: average treatment effect
BMSD: basic minimum set of data
CATE: conditional average treatment effect
DAG: directed acyclic graph
ICS: Catalan Institute of Health
PADRIS: data analysis programme for research and innovation in healthcare
PJI: periprosthetic joint infection
RCT: randomized controlled trial
RWD: real‐world data
RWE: real‐world evidence
STROBE: STrengthening the Reporting of OBservational studies in Epidemiology
TKA: total knee arthroplasty

## INTRODUCTION

Knee prosthesis survival is defined as the time elapsed between a primary arthroplasty and a revision surgery, and it is one of the most important success or failure indicators of knee arthroplasty procedures. This is so because revision surgeries are performed when a problem is discovered in the prosthesis, and that usually implies a big negative impact on the quality of life of patients [6]. Thus, prosthetic survival and the factors that influence it are two of the most widely analysed outcomes in the literature on knee arthroplasty. Researchers and clinicians aim to discover what can increase the life of the prostheses [7, 24]. One of the actions that could potentially have this effect is using antibiotic‐loaded bone cement (ALBC) during primary surgery. The effects of this intervention have long been discussed in the literature, and the evidence is considered not conclusive: different studies find beneficial effects [14], detrimental effects or no effects at all [3, 18]. For this reason, studies at all levels of evidence quality (observational studies [3, 14], randomized trials [12, 17] and systematic reviews with meta‐analysis [19, 29]) keep being published.

The survival function and the hazard ratio are two of the most widespread indicators of the correlation between ALBC and prosthetic survival in observational studies. When researchers analyse these quantities using observational data, they often avoid making explicit causal interpretations of the results [14], such as saying that ALBC *causes* an increase in prosthetic survival (although some do it implicitly [3]). This corresponds to a traditional vision, by which no causality should be inferred from observational data [20]. In recent years, both theoretical and empirical works have proven that this vision has severe limitations, and it is changing thanks to the development of causal inference [9, 10, 25]. With the causal inference approach, the causal nature of the research question can be explicitly acknowledged, even when using observational data. Causal methods provide a set of assumptions, and they guarantee that if those assumptions hold, the measured correlations are in fact causal relationships. Furthermore, empirical evidence shows that these methods can eliminate or reduce biases from observational studies in ways that traditional approaches cannot do [11, 22]. For these reasons, causal inference is being adopted in many clinical domains [23, 27, 32].

The question of whether ALBC can increase prosthetic survival is a causal one. Yet, to the best of the authors' knowledge, it has never been addressed with causal inference methods. Furthermore, these methods have little or no penetration in the literature on knee arthroplasties. As previously justified, this constitutes a limitation.

The goal of this study was to answer the question of what is the effect of ALBC on prosthetic survival using causal inference methods and data from the Catalan arthroplasty register. The hypothesis was that a positive effect would exist, based on previous analyses [8]. The secondary goal was to show some of the benefits of these methods in comparison with the traditional approach, and to serve as an introduction to the causal methodology in the literature of knee arthroplasties.

## MATERIALS AND METHODS

The current study is a retrospective cohort study. The intervention of interest was the usage of ALBC versus the alternative of using plain cement. The outcome of interest was prosthetic survival, and the considered event was the revision for any cause, that is, a surgery after the primary surgery where at least one component is revised (excluding the patellar component). The causal research question was what is the effect of using ALBC on prosthetic survival, compared to using plain cement?

**Figure 1:**
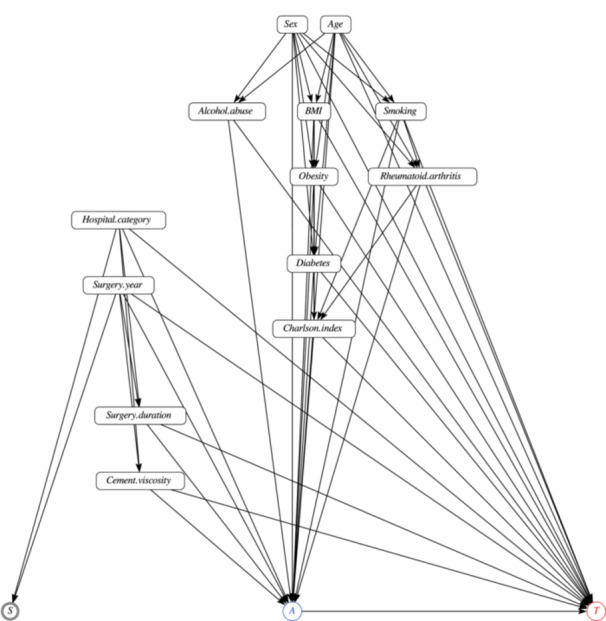
Directed acyclic graph of the problem. *A* is the treatment variable, which is a binary variable indicating whether antibiotic‐loaded bone cement or plain cement was used. *T* is the outcome variable, indicating the survival time of the prostheses. The rest of the variables are confounders. After controlling for the confounders, no biasing paths are open, and the treatment effect is estimable from the observational data.

Firstly, directed acyclic graphs (DAGs) were used for encoding expert knowledge about the problem [25]. DAGs are node‐and‐arrow schemes where nodes represent variables, and arrows represent causal relationships. Figure 1 shows the DAG of the problem, which was developed in collaboration with knee surgeons of the Parc Tauli hospital. The treatment variable, ALBC or plain cement, is represented as *A*, and the outcome variable, prosthetic survival time, as *T*. A node for potential selection bias was also included (*S*). The rest of the considered variables, explained in the Data section, are confounders, that is, they influence both the treatment and the outcome. Potential causal relationships among confounders (for instance, age may affect the body mass index [BMI], smoking status, obesity, etc.) were also represented. Note that, on the one hand, DAGs serve to explicitly represent the knowledge about the causal relationships among the variables of the problem, which allows for an open discussion about them. On the other hand, DAGs also serve for checking the identifiability of the causal query, that is, whether the causal question of interest is answerable from observational data [11, 25]. Further technical details about the identifiability of the causal query are explained in Supporting Information: File S3.

Secondly, the causal estimands were defined. Those correspond to the statistical quantities that answer the causal questions of interest. The average treatment effect (ATE) was used for the general population and the conditional average treatment effect (CATE) for subgroups, which are common estimands in clinical studies with causal inference methods [4, 11]. In particular, the ATE was defined as the average prosthetic survival probability difference between the strategy of using ALBC and the strategy of using plain cement, throughout the study time. The CATE was defined similarly, but for specific subgroups of the population represented by specific values of the confounders. The mathematical definitions of these quantities are provided in Supporting Information: File S3.

Lastly, the estimator was selected, that is, the statistical model or method for computing the estimands. In particular, causal survival forests (CSF) [21] were used, which are a method for the estimation of potentially heterogeneous treatment effects in survival settings with right censoring. This estimator works by fitting several models using Random Forests and then combining them into an estimation formula. Several features make CSF the optimal choice for this particular problem: (1) the method combines Random Forests, which are a powerful and flexible machine learning algorithm for modelling, with survival estimation; (2) it is one of the few options that explicitly accounts for censoring, by modelling the censoring process; (3) it shows top performance for heterogeneous treatment effect estimation, making it the best option for computing treatment effects in subgroups of the population. Note that heterogeneous treatment effects are those that depend greatly on patient characteristics, which is plausible in the current scenario. In comparison to CSF, traditional methods such as the Cox proportional hazards model impose stronger assumptions on the data, and perform worse in the presence of censoring. Further technical details of this method are provided in Supporting Information: File S3.

## DATA

Data from the Catalan public healthcare system were used for this study. In particular, the employed registries were the Catalan Arthroplasty Register (RACat) and several datasets from the Catalan Institute of Health (ICS) and the Catalan Public Insurer (CatSalut), such as the dataset of hospital discharges and some databases about primary care. These databases were analysed in a previous work that followed a traditional approach [8]. The main source of the data was RACat [2]. This national, population‐based registry gathers information about knee and hip arthroplasties. The dataset of hospital discharges and the databases of primary care contain information about hospital activity and primary care assistance, such as diagnoses and procedures.

**Figure 2:**
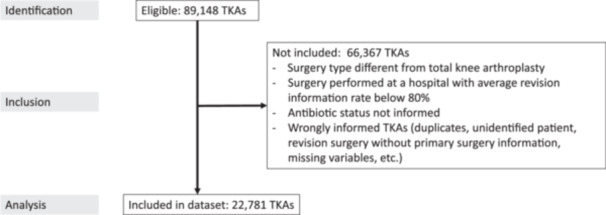
Data diagram of the study data. Starting point of 89,148 eligible total knee arthroplasties (TKAs), finally including 22,781 total knee arthroplasties in the dataset.

The information starting point was all the knee arthroplasties contained in the RACat database with surgery date between January 1, 2011 and December 31, 2020. They were followed up until December 31, 2023. Partially cemented arthroplasties and unicompartmental arthroplasties were discarded, keeping only total knee arthroplasties (TKAs). In addition, TKAs coming from hospitals with a low percentage of informed procedures were discarded, to minimize biases (see Supporting Information: File S3 for a more detailed explanation). Finally, TKAs with wrong or incomplete information were filtered. This process resulted in 22,781 TKAs for final analysis. Figure 2 shows the STROBE [31] flow diagram of this process.

Besides the usage of ALBC or plain cement and the prosthetic survival, other variables included for analysis were patient's age, sex assigned at birth, obesity, diabetes, rheumatoid arthritis, alcohol abuse, smoking status, BMI, Charlson comorbidity index, Elixhauser index, hospital category, primary surgery year and surgery duration. See Supporting Information: File S3 for further details.

The data were extracted, linked and analysed within the Data Analysis Programme for Research and Innovation in Healthcare (PADRIS), managed by AQuAS, the Agency for Health Quality and Assessment of Catalonia. National ethics committee approval with number PR186/19 and Advisory Committee of RACat approval were obtained for this study.

## RESULTS

There were 15,924 (69.9%) females and 6857 (30.1%) males in the dataset. The average age was 72.14 years (with a standard deviation of 7.73 years). In 9656 (42.4%) TKAs, plain cement was used, and in 13,125 (57.6%), ALBC was used. In the ALBC group, gentamicin was used in 12,703 (96.78%) cases, tobramycin in 410 (3.12%) cases and erythromycin in 12 (0.09%) cases. A table with all the confounder variables' values stratified by cement type can be found in Supporting Information: File S2.

### ATE

At a horizon of 120 months (10 years), this difference is 0.08 (confidence interval: 0.072–0.089) or 8 percentage points, in favour of the ALBC.

**Figure 3:**
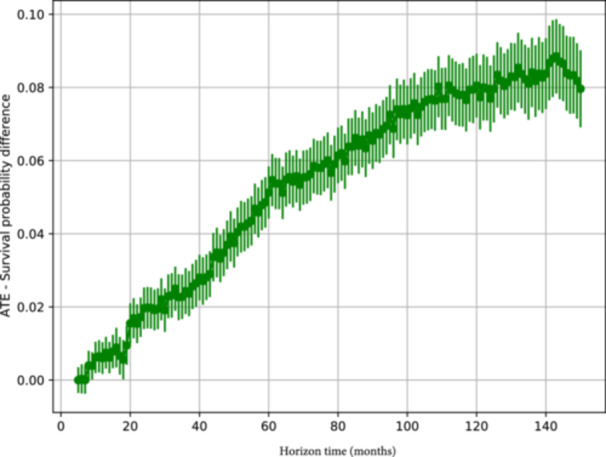
Average treatment effect (ATE), as the difference in prosthetic survival probability (*y* axis) between antibiotic‐loaded bone cement use and plain cement use, along the time horizon (*x* axis).

Figure 3 shows the ATE (*y* axis) for different time horizons (*x* axis). Point estimates are provided together with confidence intervals. The effect is small for short time horizons, gradually increases, reaching a difference of 0.05 after 50 months, 0.075 around 100 months and a maximum difference of 0.08 around 120 months. The drop that can be observed after 130 months is an effect of the employed method when the time horizon is too large for the characteristics of the analysed data (for more details about this, see Supporting Information: File S3).

### CATE

Besides the ATE, the present study aimed to calculate the CATE, that is, the effect of ALBC on prosthetic survival in the different subgroups of the population defined by the confounders. Table 1 shows the values of CATEs of the most relevant confounders at 120 months of the time horizon. The confounders whose values most depart from the ATE at 120 months are highlighted because those were the factors that had a bigger impact on prosthetic survival. Rows in green (and red) show the CATEs whose confidence intervals are above (and, respectively, below) those of the ATE and do not overlap with them. Figures with CATE values along the whole horizon‐confounder range can be found in Supporting Information: File S1.

**Table 1:** Table of CATEs of the most relevant confounders, at horizon time *h* = 120 months.

	CATE	95% CI	
Sex		
Male	0.082	(0.0811, 0.0835)	
Female	0.076	(0.0747, 0.0764)	
Age			
(28.9,32.2]	0.093	(0.0911, 0.0946)	
(32.2,35.5]	0.08	(0.0793, 0.0811)	
(35.5,38.8]	0.094	(0.0934, 0.0954)	
(38.8,42.1]	0.099	(0.0969, 0.1005)	
(42.1,45.4]	0.102	(0.1000, 0.1030)	
(45.4,48.7]	0.104	(0.1020, 0.1054)	
(48.7,52]	0.101	(0.0997, 0.1030)	
(52,55.3]	0.102	(0.1005, 0.1041)	
(55.3,58.6]	0.1	(0.0987, 0.1021)	
(58.6,61.9]	0.092	(0.0906, 0.0936)	
(61.9,65.2]	0.077	(0.0763, 0.0785)	
(65.2,68.5]	0.076	(0.0749, 0.0767)	
(68.5,71.8]	0.072	(0.0709, 0.0727)	
(71.8,75.1]	0.071	(0.0699, 0.0714)	
(75.1,78.4]	0.072	(0.0715, 0.0730)	
(78.4,81.7]	0.072	(0.0708, 0.0722)	
(81.7,85]	0.069	(0.0685, 0.0698)	
(85,88.3]	0.067	(0.0665, 0.0673)	
(88.3,91.6]	0.064	(0.0630, 0.0643)	
Obesity			
No	0.077	(0.0759, 0.0778)	
Yes	0.083	(0.0817, 0.0841)	
Rheumatoid arthritis			
No	0.077	(0.0764, 0.0784)	
Yes	0.086	(0.0848, 0.0874)	
Diabetis			
No	0.077	(0.0765, 0.0784)	
Yes	0.078	(0.0771, 0.0793)	
Smoking status			
Nonsmoker	0.076	(0.0752, 0.0771)	
Former smoker	0.081	(0.0868, 0.0895)	
Smoker	0.088	(0.0804, 0.0825)	
Alcohol abuse			
No	0.077	(0.0765, 0.0784)	
Yes	0.095	(0.0933, 0.0963)	
Viscosity			
Unknown	0.055	(0.0547, 0.0562)	
Low	0.055	(0.0546, 0.0563)	
Medium	0.081	(0.0806, 0.0819)	
High	0.13	(0.1284, 0.1317)	

*Note*: Highlighted with green (respectively, red) are CATEs whose confidence intervals are above (respectively, below) and do not overlap with the confidence intervals of the ATE at *h* = 120.

Abbreviations: ATE, average treatment effect; CATE, conditional average treatment effect; CI, confidence interval.

Table 1 shows that ALBC has a bigger positive effect for prosthetic survival probability for men (0.082) than for women (0.076), and that the effect is, in general, bigger for younger patients than for older ones (except for the very first age groups). The treatment effect was bigger for patients who had obesity (0.083 vs. 0.077) and for patients who had rheumatoid arthritis (0.086 vs. 0.077) than for patients who did not present these conditions. The same was observed for confounders such as smoking (0.076, 0.081, 0.088 for nonsmokers, former smokers and smokers, respectively) and alcohol abuse (0.077 vs. 0.095 for nonabusers and abusers, respectively). In the case of diabetes, no statistically significant difference was observed (0.077 vs. 0.078).

### Relevance of using causally explicit methods

The secondary goal of this study was to empirically show some of the benefits of the causal approach for answering the research question about the effect of ALBC on prosthetic survival. For this purpose, a side analysis was conducted under the hypothesis that, in Catalonia, the usage of ALBC or plain cement was determined solely by the hospital category, which would be a plausible scenario under certain protocols. Thus, a new DAG was developed to reflect the aforementioned hypothesis, and the ATE was computed in that scenario. The obtained ATE differed from the one calculated in the original scenario. This validates the idea that making causal assumptions explicit is crucial because it impacts the estimated quantities and shows the strength of causal methods for making assumptions explicit and operating under different sets of them. The details about this experiment can be found in Supporting Information: File S3.

## DISCUSSION

The main findings of this study were that ALBC increased prosthetic survival, both in the whole population as well as in subgroups defined by the confounders, and that the causal inference framework and its methods showed their benefits for tackling the research question.

### Prosthetic survival in the overall population

In the present causal study, ALBC showed a protective effect, increasing prosthetic survival. This effect was small for short horizon times and grew until stabilizing around a value of 8 percentage points after 120 months.

It is well known that the literature contains works with evidence in favour of ALBC, works that found no differences between strategies, and even fewer works with evidence against ALBC [3, 14, 18]. This study's results align with the former. Individually discussing most previous studies is avoided, as this has already been done several times in the literature. Nevertheless, the works by Leta et al. [18] and Li et al. [19] are commented on, as they are two of the most modern evidence aggregation studies about this topic, with the largest analysed sample sizes. The former is a multiregistry study with almost 2 million TKAs and information coming from several countries. Individual register results were aggregated, and no statistically significant differences between ALBC and plain cement groups were observed for the analysed outcomes. Similarly, the latter is a classical systematic review with meta‐analysis encompassing more than 2 million TKAs analysed, which includes the most relevant studies previously published. Such study did not find statistically significant differences in favour of ALBC. The most likely reason for the differences observed between the results of these works and the results of the present study is actual, real differences in the data. Nevertheless, other possible spurious explanations include hidden confounding or model‐driven bias. None of the observational works in the literature analysed the research question from a causally explicit perspective nor employed causal inference methods.

Studies across all levels of evidence quality have been published and continue to be published, including observational studies, randomized controlled trials (RCTs) and systematic reviews with meta‐analysis [14, 17, 19]. The fact that in some countries both TKA and periprosthetic joint infection (PJI) rates have increased in recent years [15] ensures that research on this topic will continue until more conclusive evidence is gathered and clinical recommendation guides are updated accordingly. Cost‐effectiveness studies [1, 13] will also play a crucial role in defining the strategy of each country or healthcare system regarding the usage of ALBC in TKA. It is also worth mentioning that many works in the literature analyse only the endpoint of septic revision [19, 30]. On the contrary, the overall prosthetic survival was analysed in the present study. Although the authors agree on the interest of analysing the effect of ALBC, particularly for infection, overall survival is a more general indicator that was of greater interest to us.

### Prosthetic survival in subgroups of the population

This study's results showed that all the subgroups of the population (defined by the confounders) benefited from ALBC, but that men, younger patients, patients who had obesity or rheumatoid arthritis and patients who abused alcohol or smoked benefited more (in effect size, i.e., prosthetic survival) than their counterparts. Although these results were not directly comparable to those provided by the traditional Cox proportional hazards model due to the fundamentally different nature of the estimated quantities, it must be highlighted that the analysis of the present work showed bigger benefits for patients who presented risk factors according to most works in the literature [18, 19, 28].

### Causal inference methods

There is a growing consensus that evidence on the effects of health interventions should come both from RCTs and observational studies with real‐world data (RWD) [5, 16, 26]. Among other reasons, this is because real‐world evidence studies (i.e., studies with RWD) can alleviate some of the limitations of RCTs, such as potentially low external validity. Generating real‐world evidence with the highest possible quality requires, besides high‐quality data, adequate techniques and methods for analysis. With respect to that, there is also a growing consensus that causal inference is the most suited approach for the task [5]. For these reasons, causal inference is becoming more common in clinical studies [27, 32]. Yet, to the best of authors' knowledge, no previous study about the effects of ALBC had taken this approach. The only partial exception was the work by Keemu et al. [15], in which a DAG of the causal effects of the variables of the problem was provided. Nevertheless, this work only analysed risk factors, without defining an intervention, and thus it is fundamentally different from the present study. It is important that the literature of TKA (and, particularly, the literature of the effects of ALBC on prosthetic survival) familiarizes itself with causal inference and its methods.

### Limitations

Regarding the data, on the one hand, it must be mentioned that RACat data completeness varied for each hospital. This was corrected by analysing TKAs performed in hospitals that had at least 80% of prosthetic revision reporting rate. On the other hand, septic revision diagnostic categories of RACat were used to identify PJI, which means that infections that were not treated surgically, either superficial or deep, were not identified by this method. It is worth noting that this limitation is shared with other studies, making results comparable in principle.

Regarding the methods, the most important limitation is the potential existence of unidentified confounders (such as potentially, surgical time, individual surgeons, individual hospitals or others) that could have introduced bias in the results. This limitation is also shared with other observational studies.

Finally, the potential practical impacts of the findings of this study, such as the number of revised prostheses, the cost for the healthcare system and so on, have not been discussed. Although the authors of this study deem it important, it falls outside the scope of the present work.

## CONCLUSIONS

The causal inference approach and methods are best suited for analysing the effect of ALBC on prosthetic survival with observational data, given that the nature of the research question is causal. The present study serves as a much‐needed introduction of these methods in the literature on arthroplasty surgery. In the current study's registries, ALBC increased prosthetic survival, both in the whole population and in sub‐groups defined by the confounders. The present piece of evidence can be integrated into future evidence aggregation studies, as this topic remains under discussion in the literature.

## AUTHOR CONTRIBUTIONS

**Borja Velasco‐Regúlez**: Conceptualization; data curation; formal analysis; methodology; software; writing—original draft. **Sergi Gil Gonzalez**: Conceptualization; investigation; validation; writing—review and editing. **Jesus Cerquides Bueno**: Conceptualization; project administration; resources; supervision; validation; writing—review and editing.

## CONFLICT OF INTEREST STATEMENT

The authors declare no conflict of interest.

## ETHICS STATEMENT

National ethics committee approval with number PR186/19 and institutional review board were obtained for this study, given the retrospective nature, and all the procedures being performed were part of the routine care. The related documentation is available if required.

## Supporting information

Supplementary figures.

Supplementary Table 1.

Supplementary material.

## Data Availability

The data employed for this study were individual patient pseudo‐anonymized data and were obtained under the Data Analysis Programme for Research and Innovation in Healthcare (PADRIS), managed by AQuAS, the Agency for Health Quality and Assessment of Catalonia. This data cannot be made available publicly.
