# Supplementary material for: The first causal inference analysis of the Catalan Arthroplasty Register shows a positive effect of antibiotic‐loaded bone cement on knee prosthesis survival
Source: J Exp Orthop. 2025 Dec 17;12(4):e70574. doi: 10.1002/jeo2.70574 (PMC12709647; doi:10.1002/jeo2.70574)

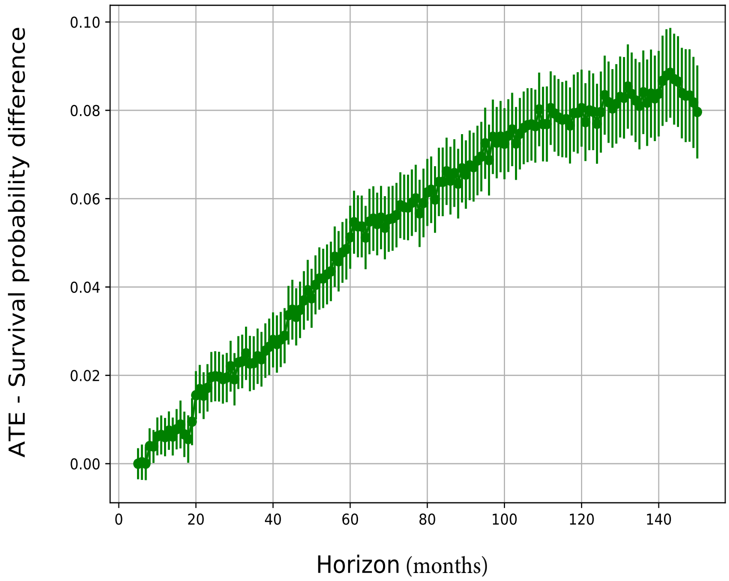

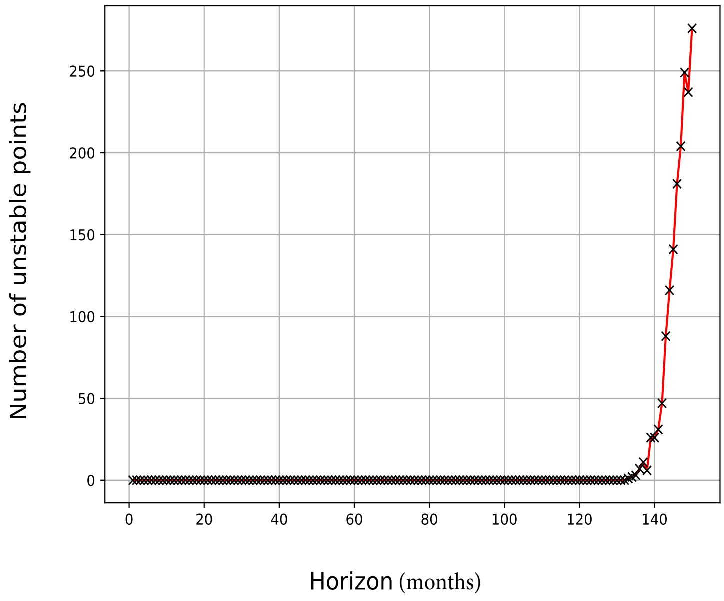

Supplementary figure 1. Top: Average treatment effect as difference in prosthetic survival probability between antibiotic-loaded bone cement use and plain cement use, along horizon time. Bottom: number of unstable datapoints along time. An unstable datapoint is an observation in the dataset that has been estimated to have extreme values of treatment and/or censoring probabilities. Unstable datapoints can introduce errors in the ATE.

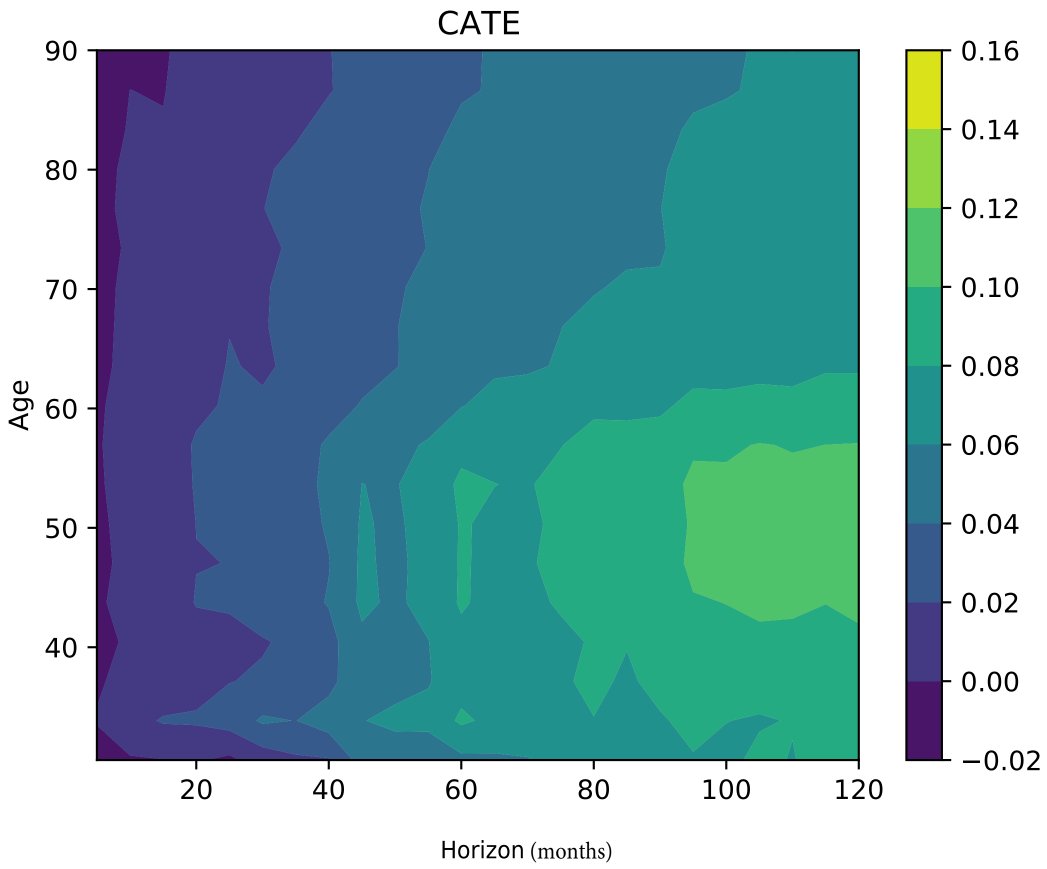

Supplementary figure 2. CATE for age. The impact of age on the CATE can be seen for medium and long time horizons, with younger patients getting a bigger benefit in prosthetic survival from using antibiotic-loaded bone cement. Thus, above 60 months important differences in the CATE between patients below 60 years and above 60 years can be observed, the former having a CATE of 7.5%-10% and the latter of 5%-7.5%. Note that we consider the values above 90 years to be less reliable due to the small sample size.

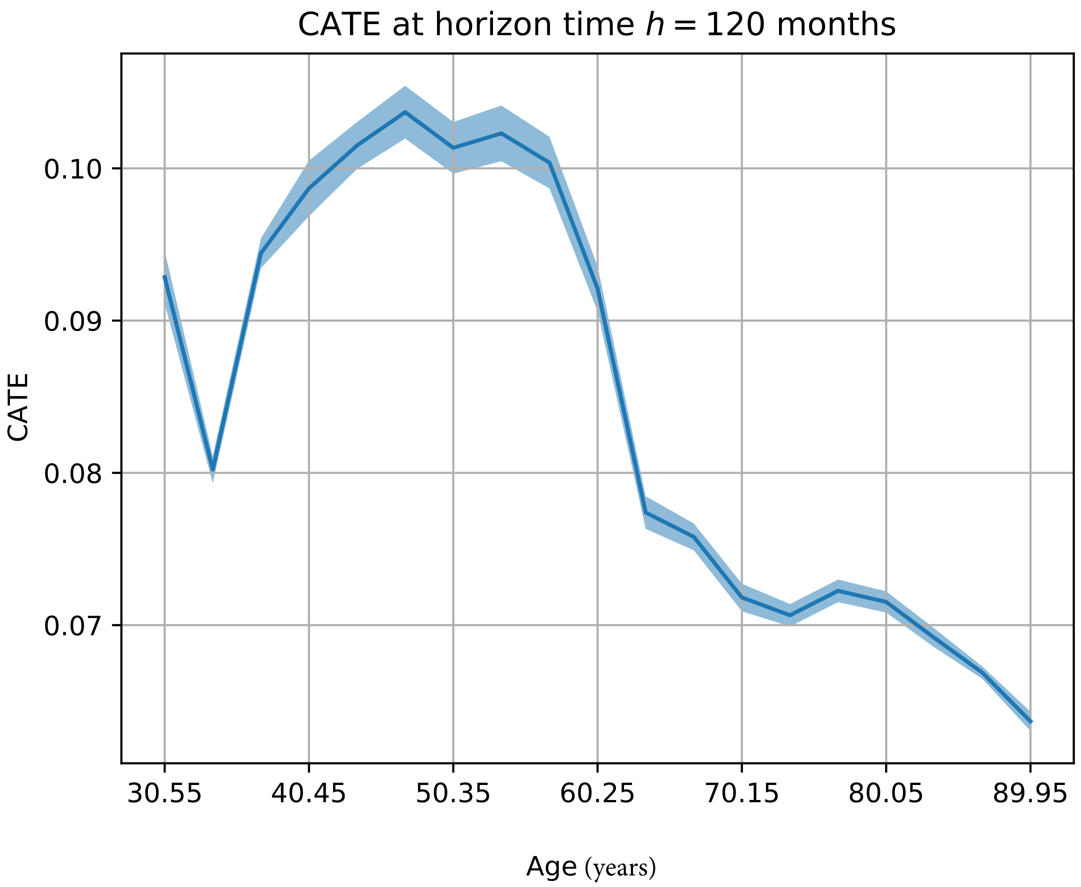

Supplementary figure 3. CATE for age, at the specific value of horizon time of h = 120 months.

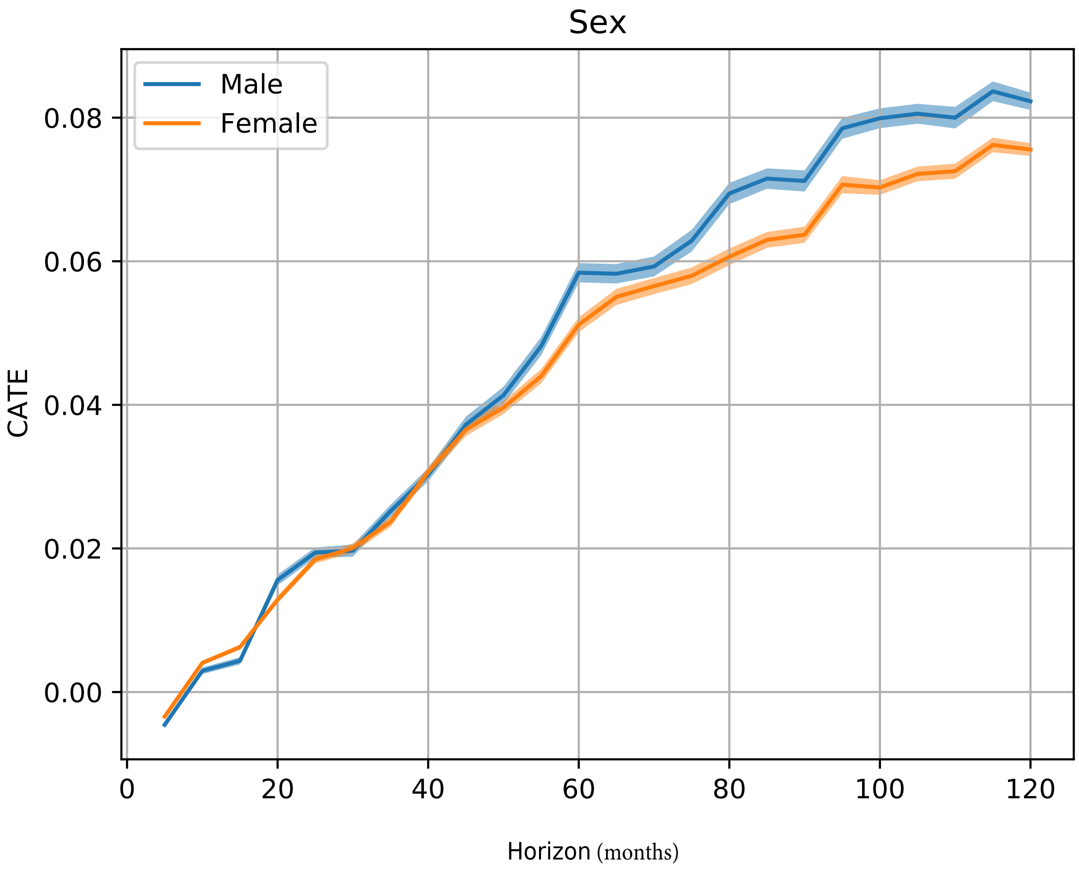

Supplementary figure 4. CATE for sex. The sex does not have an impact for short time horizons. A small effect becomes observable from 50 months on, but remains smaller than 1 percentage point difference for the rest of the study time.

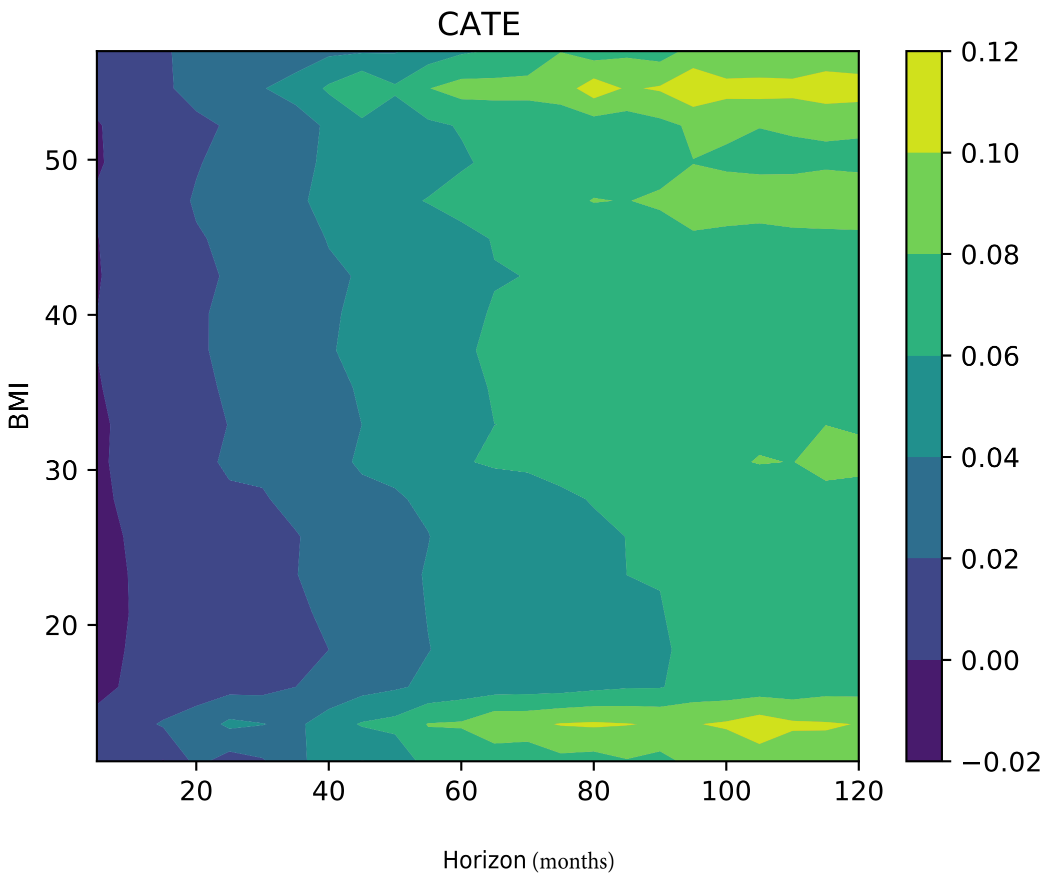

Supplementary figure 5. CATE for BMI. Patients with bigger values of BMI (above 40) benefit more from antibiotic-loaded bone cement, especially for longer horizon times. Differences range between 8%-12% for a BMI value above 40, and between 6%-8% below that value.

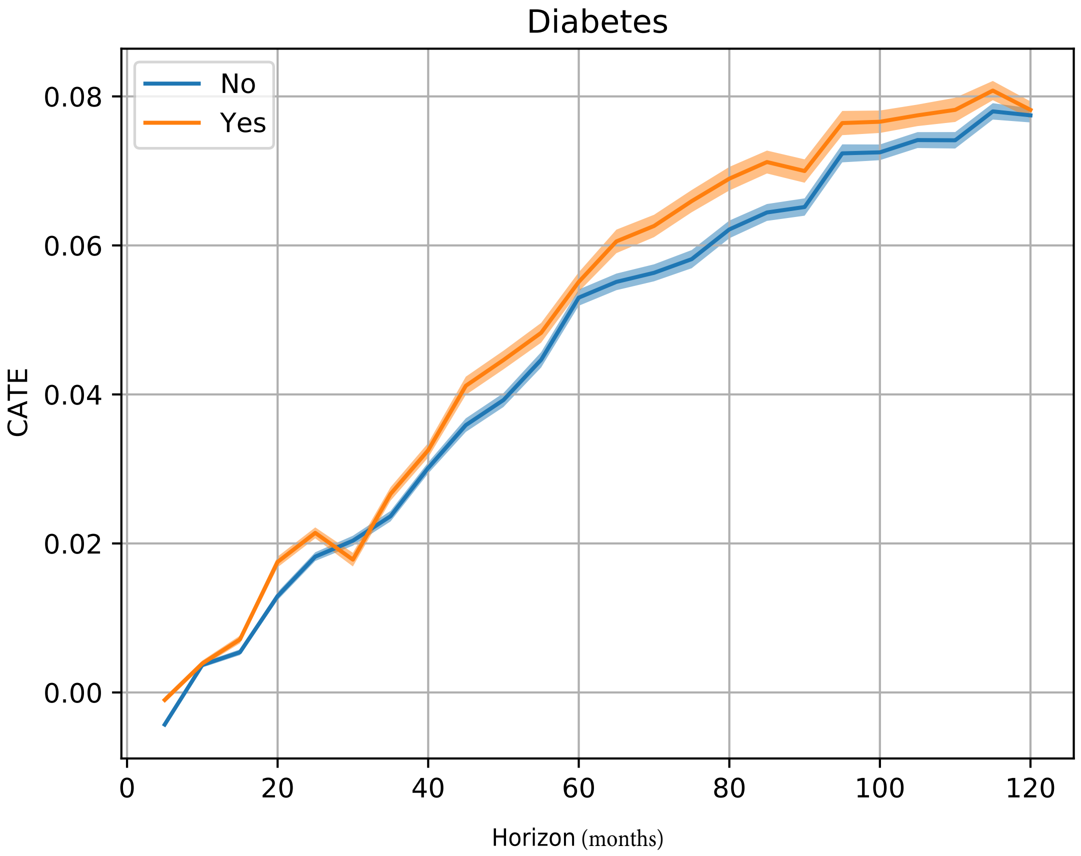

Supplementary figure 6. CATE for diabetes. Almost no differences for short time horizons that then amplify, but always remaining below 1 percentage point. Patients with the disease benefit more from antibiotic-loaded bone cement use.

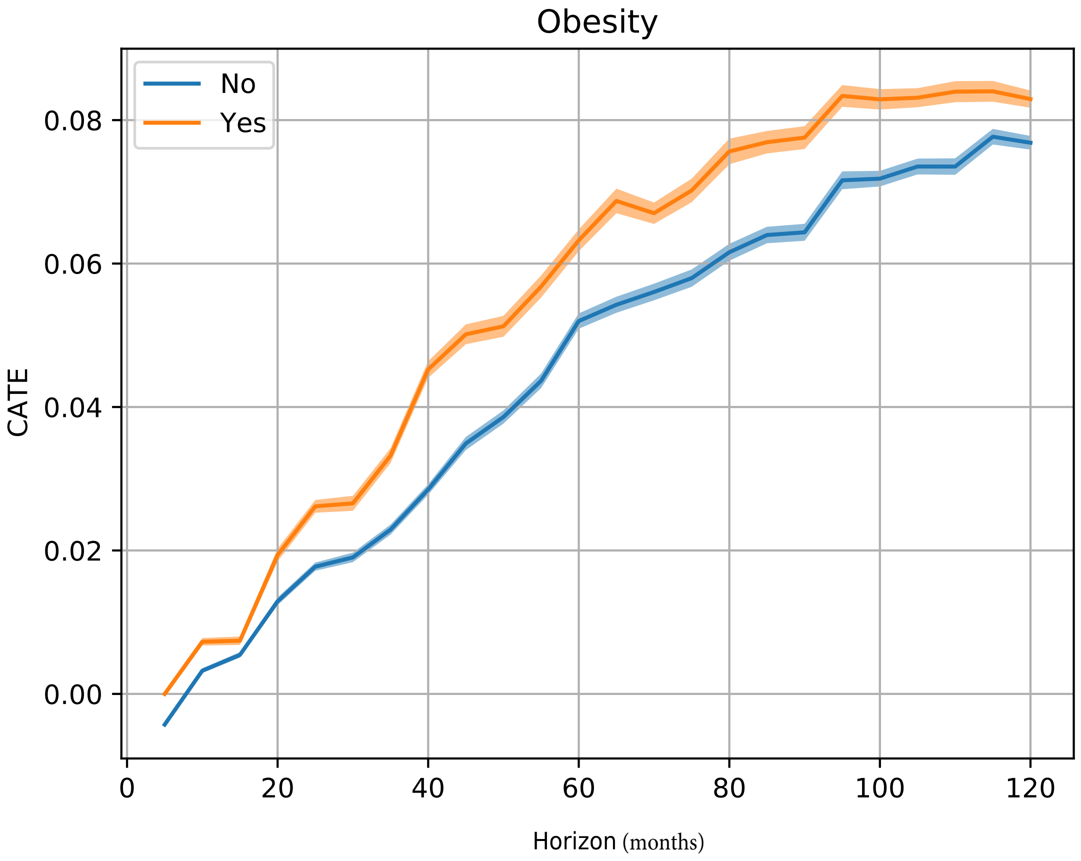

Supplementary figure 8. CATE for obesity. Small differences for short time horizons that then amplify, but always remaining slightly above 1 percentage point. Patients with the disease benefit more from antibiotic-loaded bone cement use.

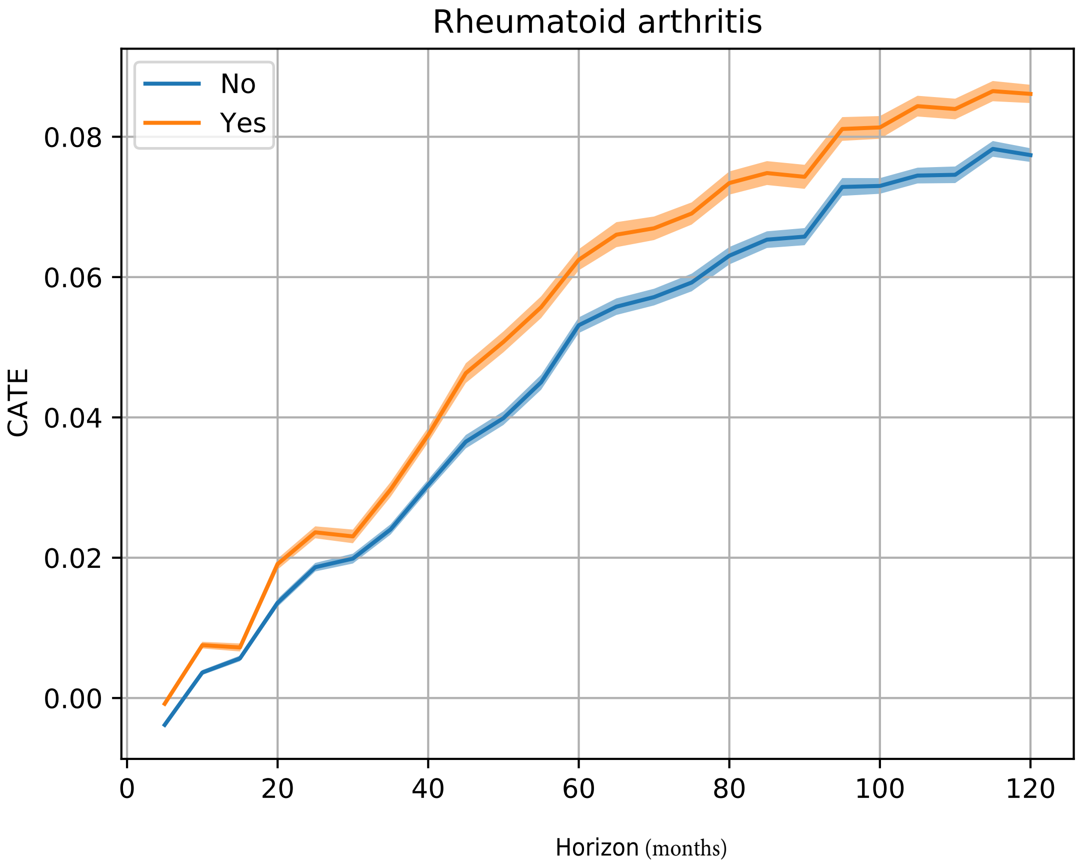

Supplementary figure 9. CATE for rheumatoid arthritis. Small differences for short time horizons that then amplify, but always remaining around 1 percentage point. Patients with the disease benefit more from antibiotic-loaded bone cement use.

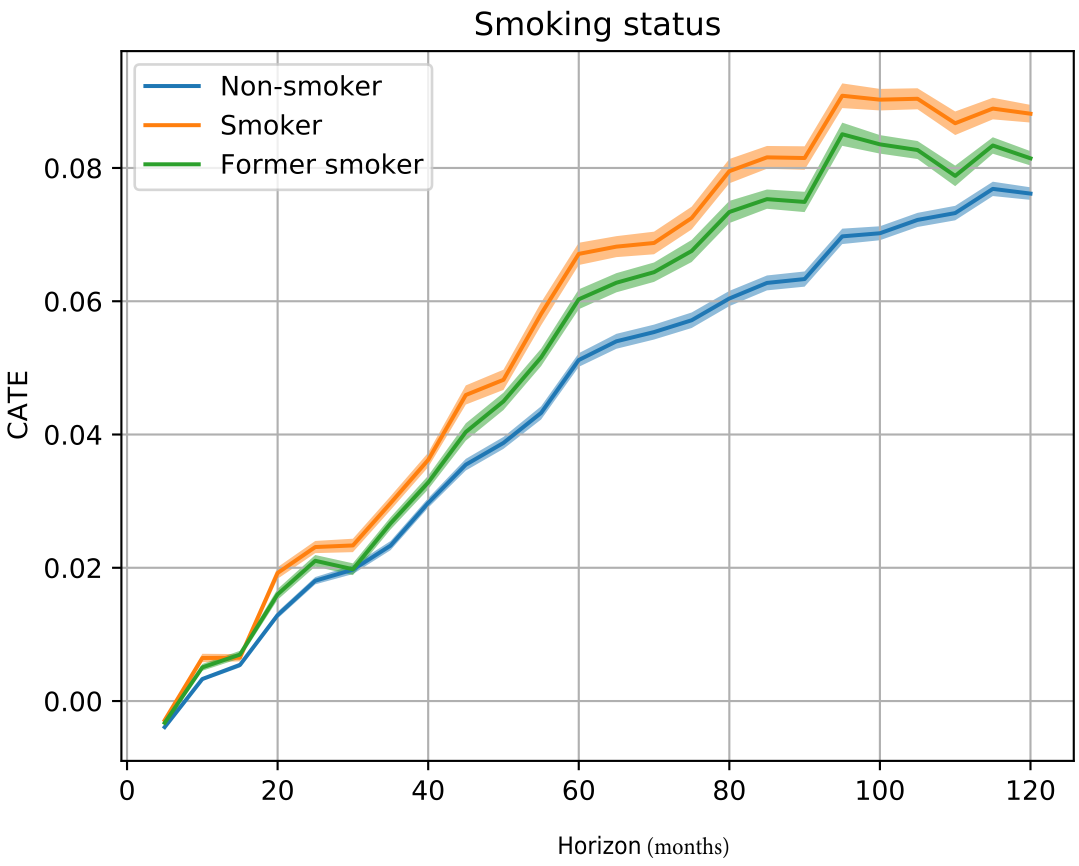

Supplementary figure 10. CATE for smoking status. 0 for non-smoker, 1 for former smoker and 2 for smoker, at the time of surgery. Patients who smoke or used to smoke benefit more from the use of antibiotic-loaded bone cement, with differences increasing with horizon time.

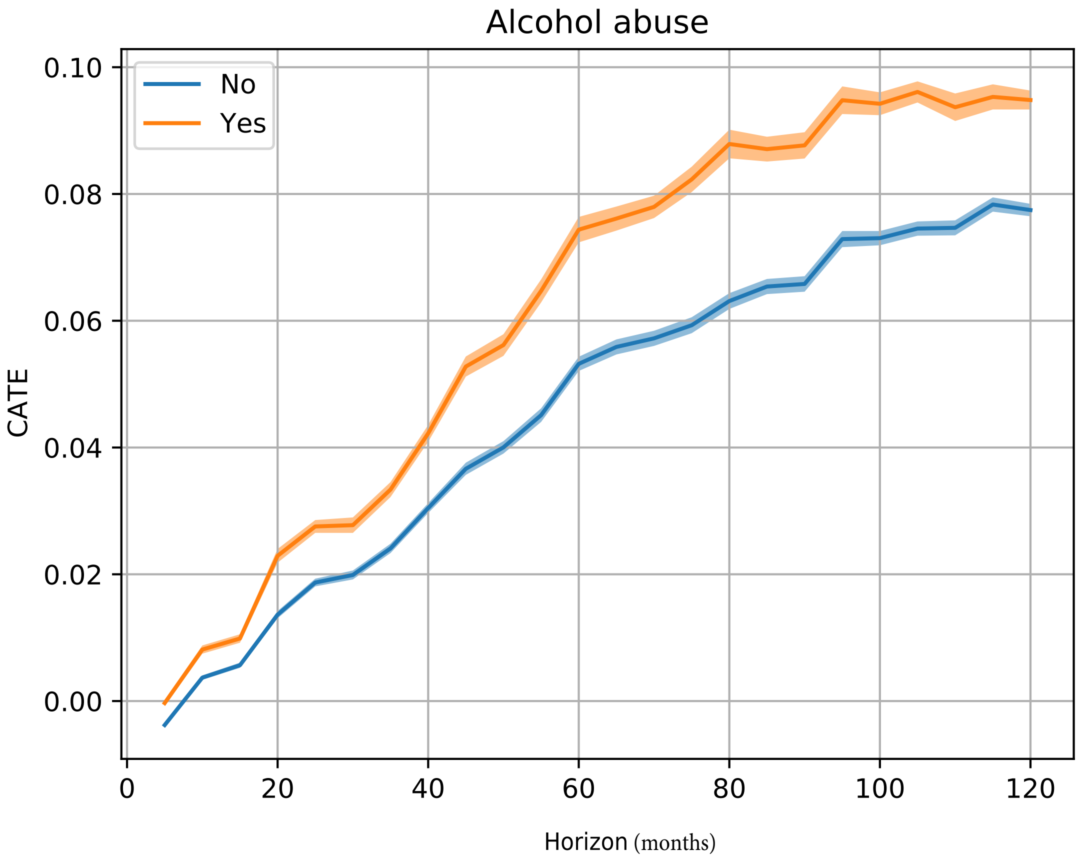

Supplementary figure 11. CATE for alcohol abuse. Patients who abuse alcohol benefit more from the use of antibiotic-loaded bone cement, differences increase with horizon time and reach values above 2 percentage points.

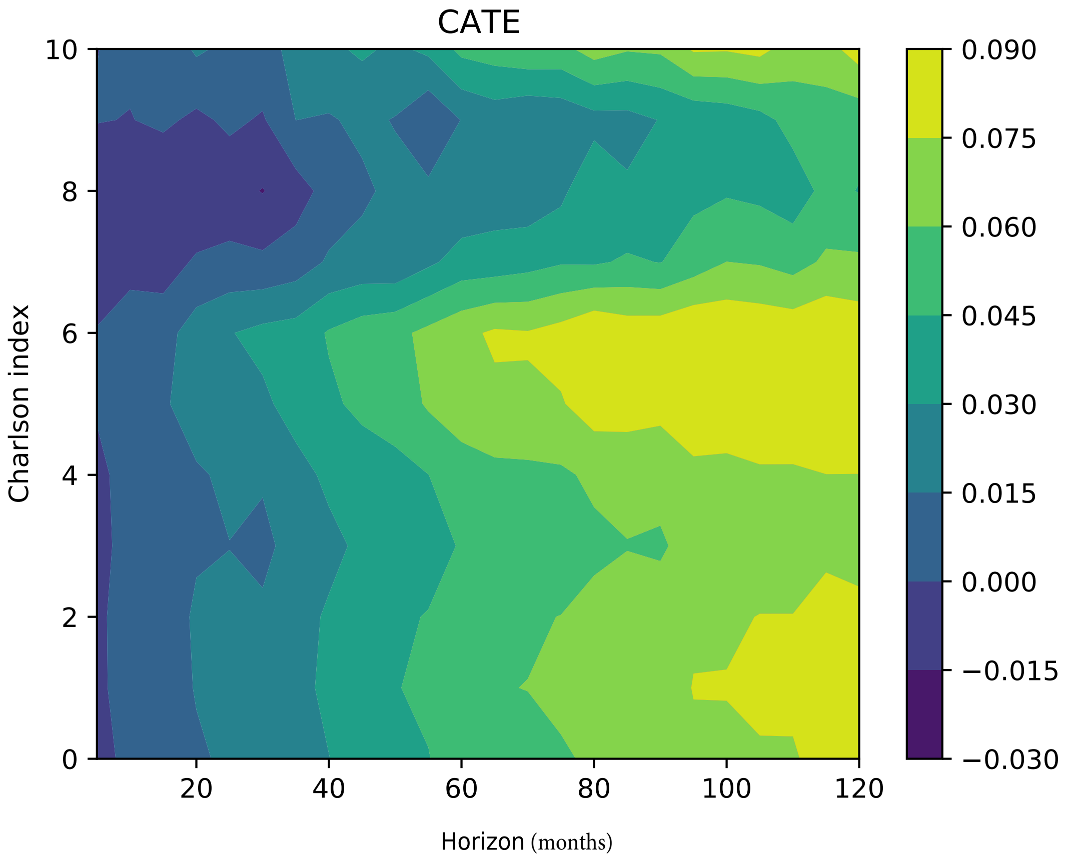

Supplementary figure 12. CATE of the Charlson index.

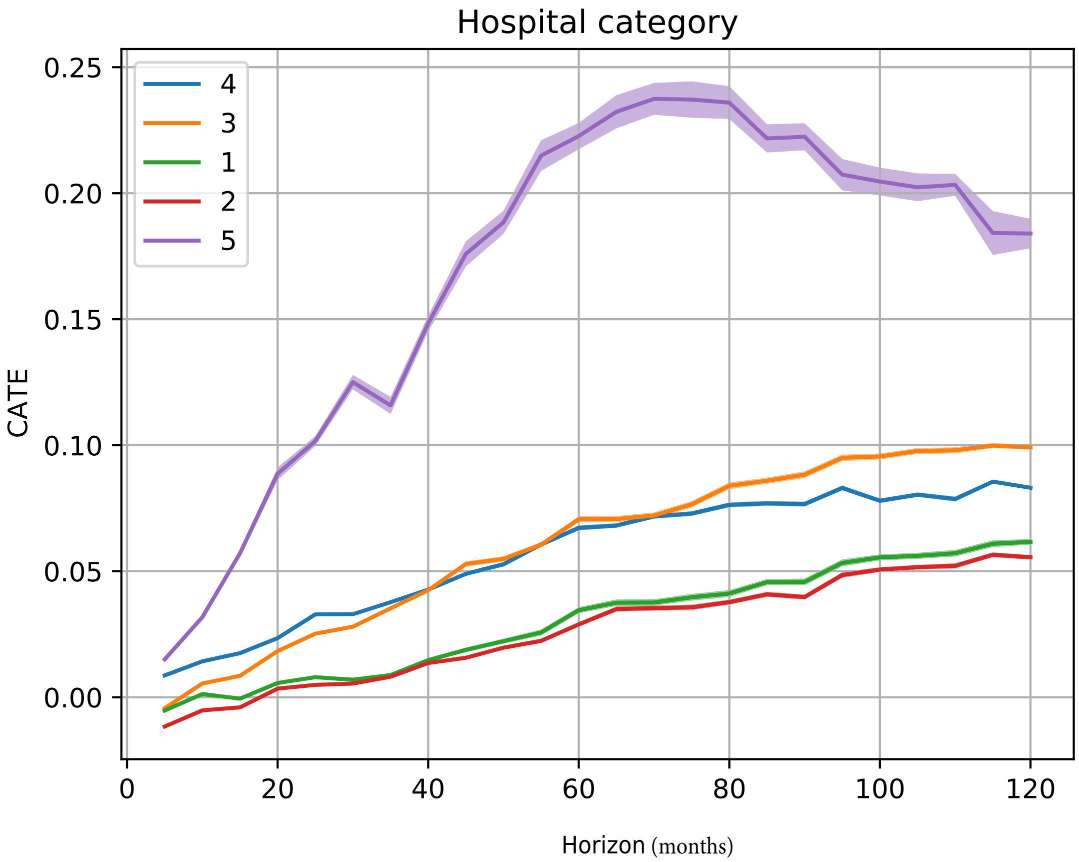

Supplementary figure 13. CATE of the hospital category.

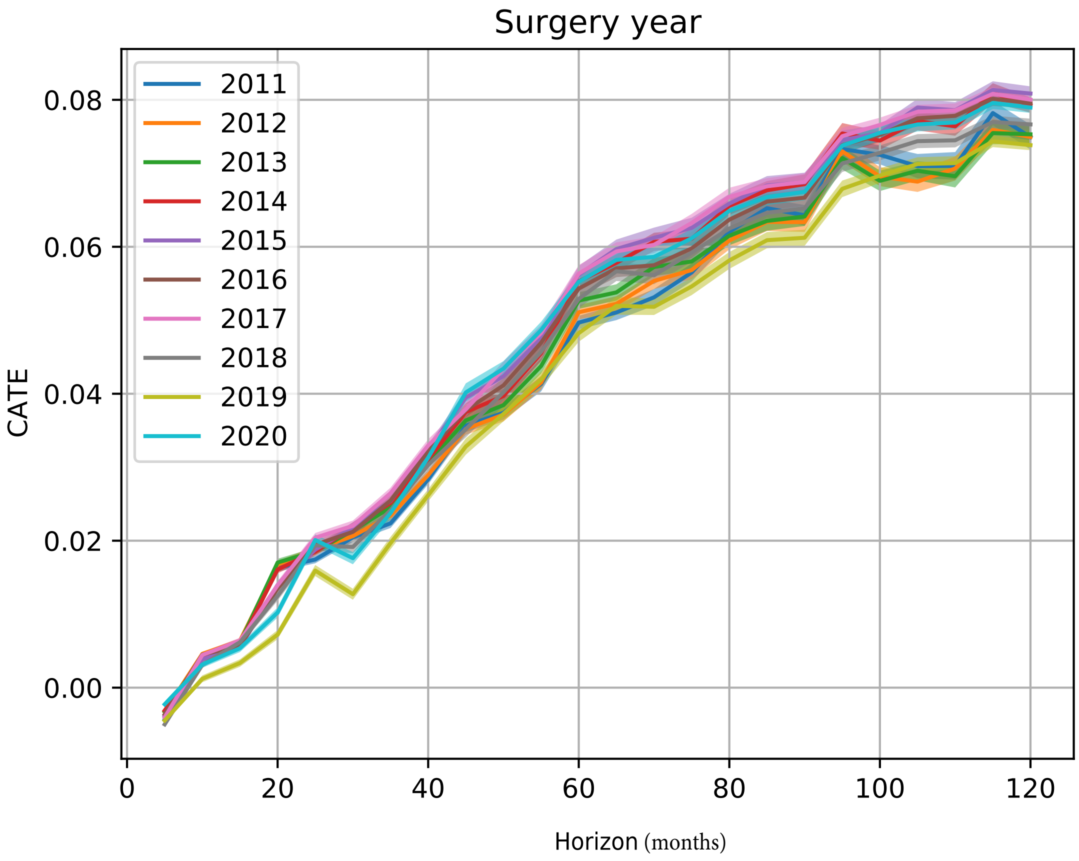

Supplementary figure 14. CATE of the surgery year.

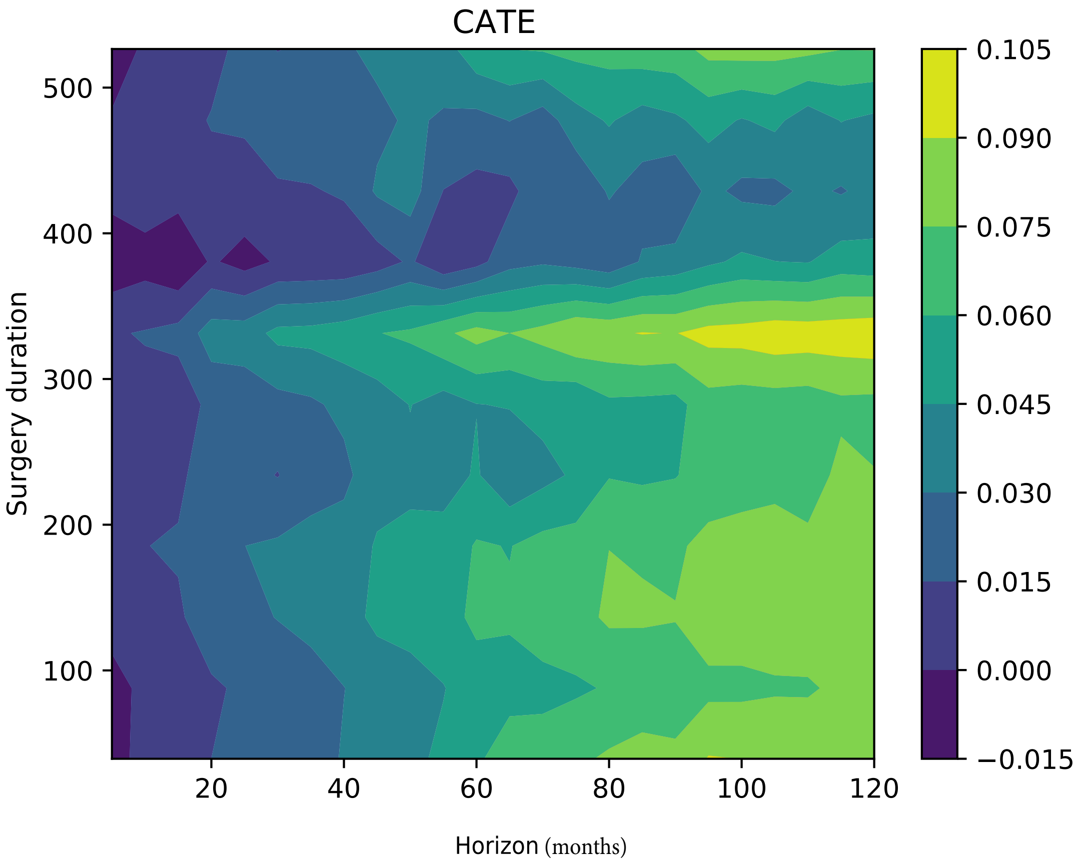

Supplementary figure 15. CATE of the surgery duration.

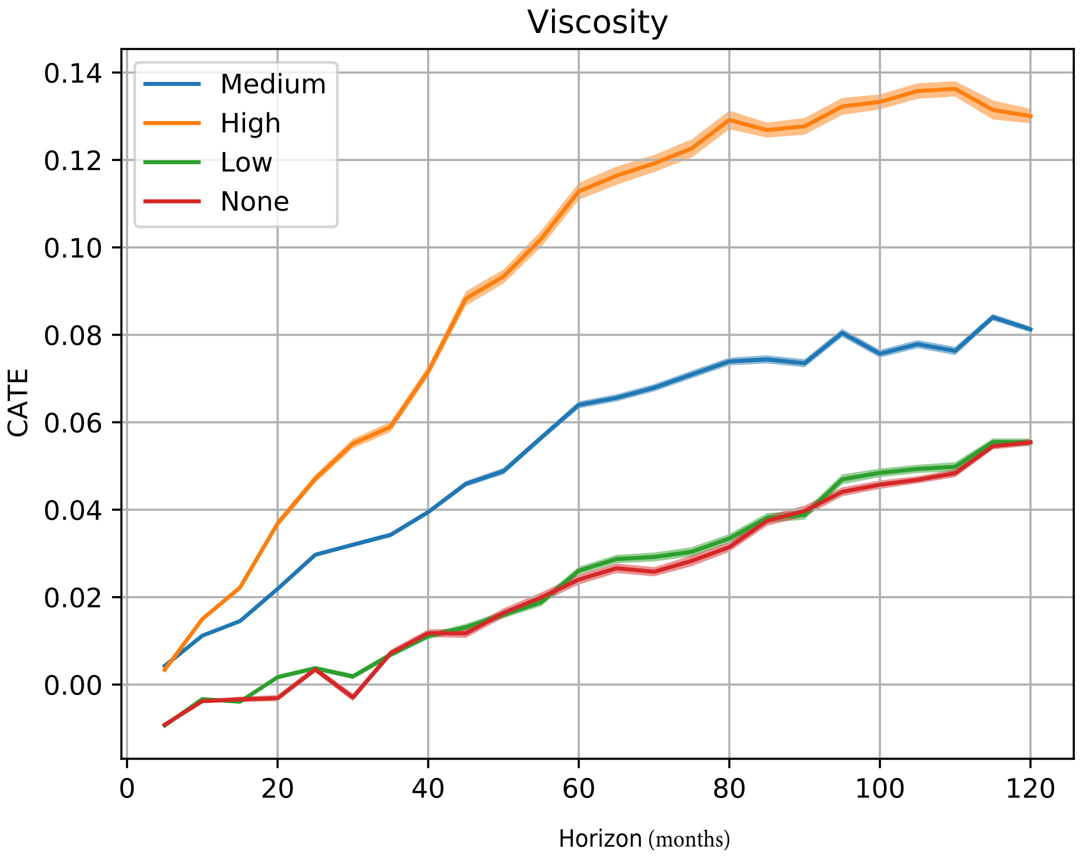

Supplementary Figure 16. CATE of the cement viscosity.

Supplementary figure 17. Hypothetical DAG, where the treatment is only influenced by the hospital category.

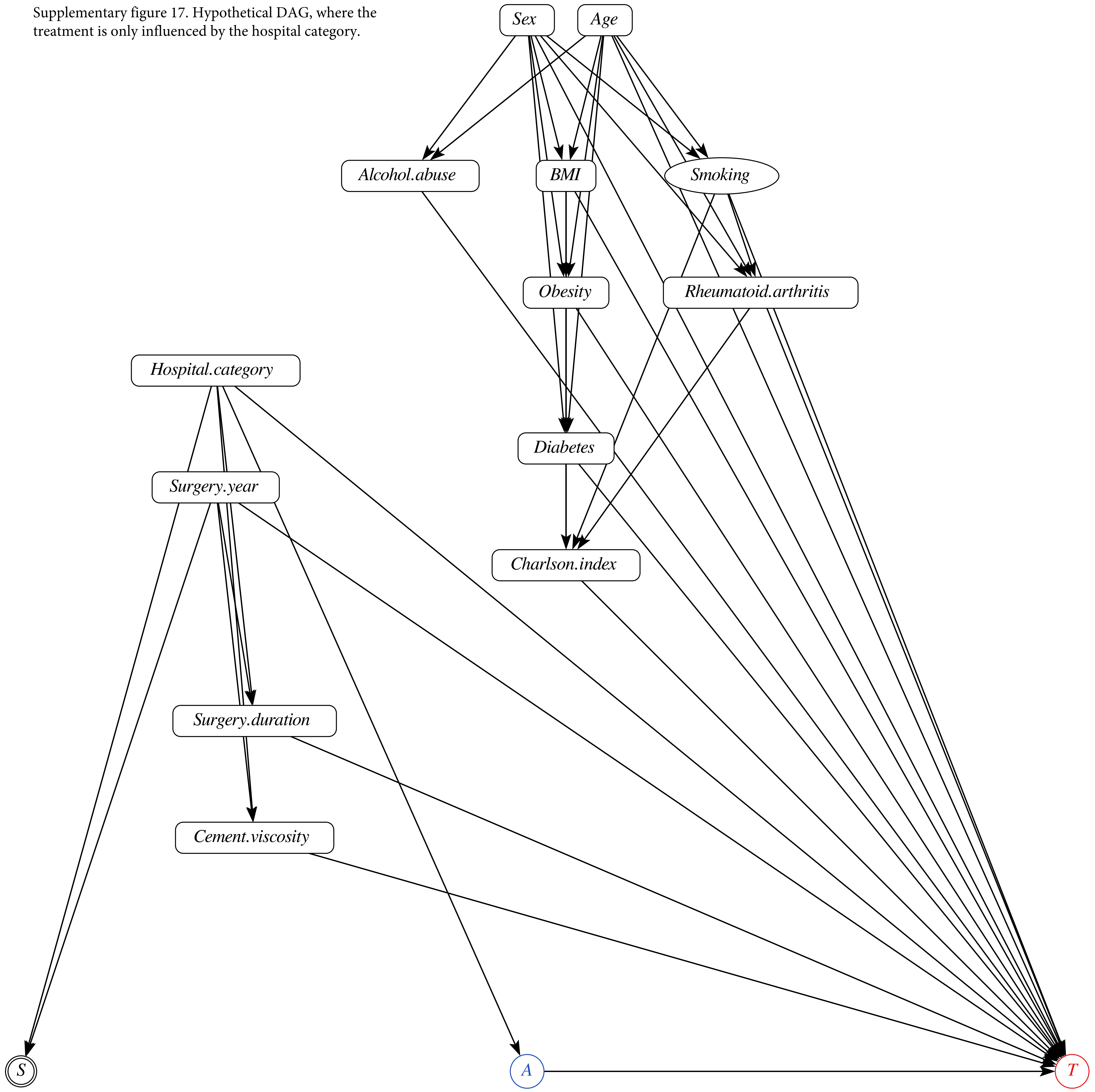

Supplementary figure 18. ATE estimates under the two different DAGs. The difference in the estimates shows the impact the DAG has, and thus the importance of its correct specification.

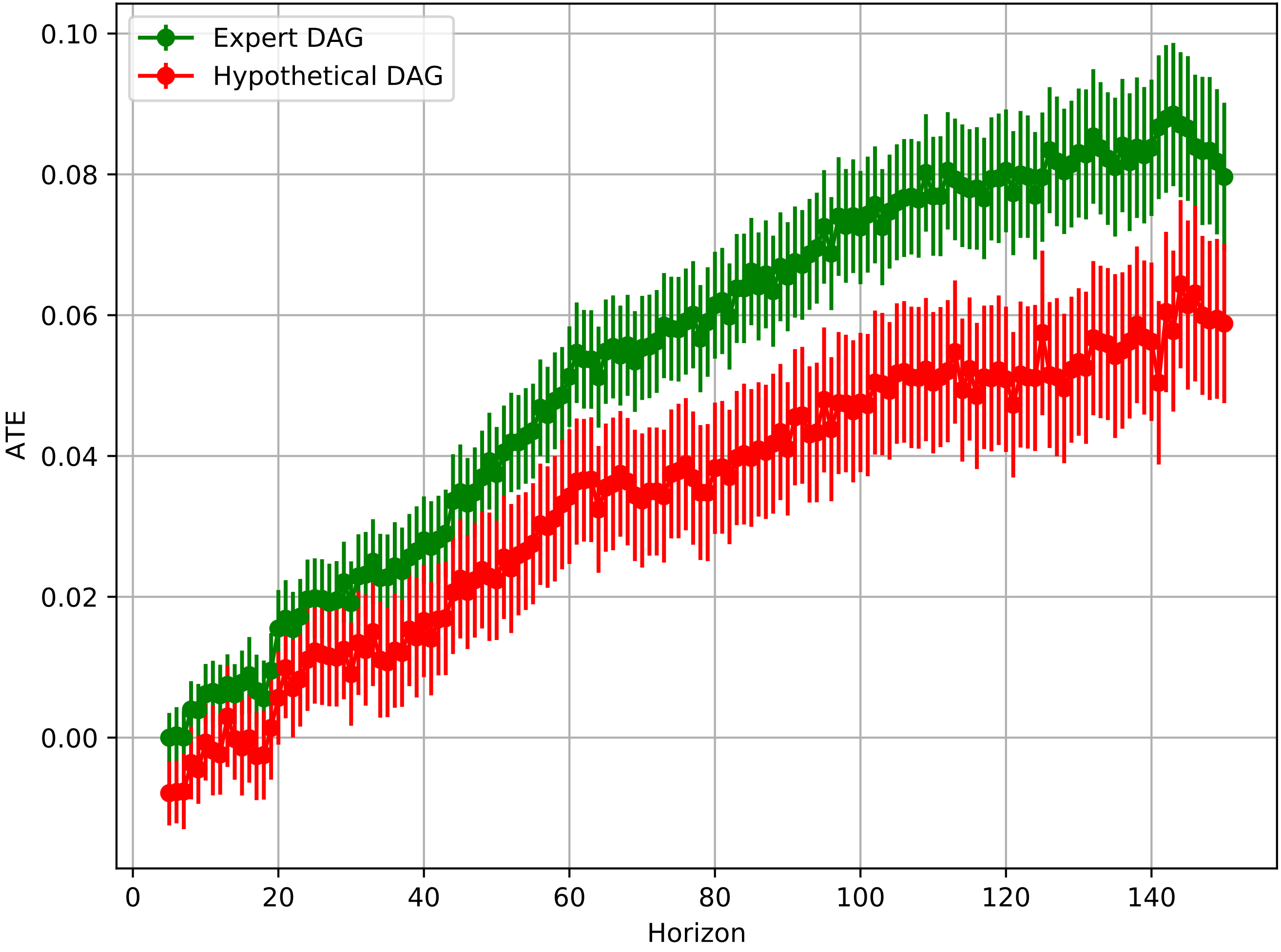

Supplement: Supplementary file 1 — Supplementary figures. [file JEO2-12-e70574-s003.pdf]
