# Supplementary material for: The first causal inference analysis of the Catalan Arthroplasty Register shows a positive effect of antibiotic‐loaded bone cement on knee prosthesis survival
Source: J Exp Orthop. 2025 Dec 17;12(4):e70574. doi: 10.1002/jeo2.70574 (PMC12709647; doi:10.1002/jeo2.70574)
Supplement: Supplementary file 2 — Supplementary Table 1. [file JEO2-12-e70574-s002.pdf]

Characteristic	Categories	Plain Cement	ALBC	P-value
		n (%)		
Sex	Female	6843.0 (42.97%)	9081.0 (57.03%)	<0.01
	Male	2813.0 (41.02%)	4044.0 (58.98%)	
		mean (SD)		
Age (years)		72.24 (7.61)	71.94 (8.01)	<0.01
Hospital Category	1	2246.0 (49.50%)	2291.0 (50.50%)	<0.01
	2	175.0 (4.20%)	3994.0 (95.80%)	
	3	3232.0 (48.56%)	3423.0 (51.44%)	
	4	3995.0 (56.32%)	3099.0 (43.68%)	
	5	8.0 (2.45%)	318.0 (97.55%)	
		mean (SD)		
Surgery duration		89.82 (14.40)	89.98 (21.21)	n.s.
Charlson Index		0.41 (0.76)	0.46 (0.79)	<0.01
Elixhauser index		1.34 (1.18)	1.41 (1.20)	<0.01
		n (%)		
Obesity	No	8509.0 (42.49%)	11518.0 (57.51%)	n.s.
	Yes	1147.0 (41.65%)	1607.0 (58.35%)	
Diabetes	No	8112.0 (42.68%)	10893.0 (57.32%)	0.04
	Yes	1544.0 (40.89%)	2232.0 (59.11%)	
Rheumatoid arthritis	No	9460.0 (42.47%)	12817.0 (57.53%)	n.s.
	Yes	196.0 (38.89%)	308.0 (61.11%)	
Alcohol abuse	No	9601.0 (42.43%)	13025.0 (57.57%)	n.s.
	Yes	55.0 (35.48%)	100.0 (64.52%)	
Smoking status	Non smoker	7985.0 (43.37%)	10426.0 (56.63%)	<0.01
	Smoker	555.0 (39.67%)	844.0 (60.33%)	
	Former smoker	1116.0 (37.56%)	1855.0 (62.44%)	
		mean (SD)		
Body mass index		31.82 (4.57)	31.76 (4.89)	n.s.

Supplementary table 1. Distribution of the confounder variables of the study population, stratified by the cement type.
