# Supplementary material for: The first causal inference analysis of the Catalan Arthroplasty Register shows a positive effect of antibiotic‐loaded bone cement on knee prosthesis survival
Source: J Exp Orthop. 2025 Dec 17;12(4):e70574. doi: 10.1002/jeo2.70574 (PMC12709647; doi:10.1002/jeo2.70574)
Supplement: Supplementary file 3 — Supplementary material. [file JEO2-12-e70574-s001.docx]

**SUPPLEMENTARY MATERIAL**

**Sensitivity analysis of revision reporting rate**

In order to compute the percentage of informed procedures for each hospital, we linked the RACat database and the BMSD database, requiring an exact match for patient identifier, surgery type and date of surgery. Then, the procedure information rate was computed as the fraction of procedures registered by RACat with respect to those registered by BMSD, which is of mandatory registration. We set the threshold in 80% of informed procedures. We performed a sensitivity analysis with respect to this parameter (percentage of revision reporting), ranging from 75 to 95% in 5% increases, and analyzed the total rate of revisions and total rate of infection in each scenario. We observed that increasing this parameter increased the percentage of revised prostheses as well as the percentage of infected prostheses, as should be expected. For the final analysis, we set the average percentage of revision rate to 80%. The choice was a tradeoff between having as many TKAs as possible (achieved by lowering the percentage of revision rate), and minimizing the potential bias derived from losing revisions (achieved by increasing the percentage of revision rate). The selected rate was also the one recommended by the RACat database managers.

**General definitions for the employed methods**

Let A be the treatment, antibiotic-loaded bone cement use or plain cement use, expressed as a binary variable that takes values 1 or 0, respectively; let T be the time passed between surgery and event dates, a continuous time variable measured in months; let X be a vector of confounders, formed by {*Age, Sex, Hospital category, Surgery year, Cement viscosity, Surgery duration, Obesity, Diabetes, Rheumatoid arthritis, BMI, Charlson index, Smoking, Alcohol abuse*}, with each variable defined in the main text; and let S be selection bias, a binary variable that takes values 1 or 0, indicating whether a confounder influences the likelihood of a patient being present in the data base or not.

**DAG and the identifiability assumptions**

Figure 2 of the main text presents the DAG of our problem. We use a set of rules known as *do*-calculus [17] to determine whether the causal effect of the treatment *A* on the outcome *T* is identifiable from the available observational data. Another way to express this is that we aim to determine what is the minimum set of variables that we need to “control for”, for obtaining an unbiased estimate of our causal query.

Using *do*-calculus notation and the CausalFusion software, we show that the causal effect *do(A = 1)* on *T*  given *X*, written as *P (T |do(A = 1), X)* is recoverable from the distribution of observed variables with selection bias, *P (A, T, X|S = 1)*.

*Proof.*

*P* (*T* |*do*(*A* = 1)*, X*) (1)

= *P* (*T* |*A, X*) (2)

= *P* (*T* |*A, X, S* = 1) (3)

*Eq. (*[*2*](#_bookmark0)*) follows from the second rule of do-calculus with the independence (A ⊥ T |X)GA Eq. (*[*3*](#_bookmark1)*) follows from the first rule of do-calculus with the independence (S ⊥ T |A, X). Finally, we get*

*P* (*T* |*do*(*A* = 1)*, X*) = *P* (*T* |*A, X, S* = 1)

Thus, controlling for the set of confounders *X*, we can indeed recover the causal effect of interest. Note that the first assumption for this to hold is that we have correctly identified all confounder variables, which is known as the “no hidden confounding” or “conditional exchangeability” assumption.

The other two assumptions are positivity and consistency. Positivity implies that we have patients treated with both treatment options in all strata defined by confounder values. This is the only assumption that is testable from the data. Consistency implies that our intervention of interest is well defined. In-depth explanations and mathematical definitions of these assumptions can be found in the Hernan MA et al. [16].

**Definitions and details about the estimands and the estimators**

Remind that the main goal of the study is to estimate the effect of antibiotic-loaded bone cement on prosthetic survival probability, in the presence of confounders and censoring. Given the treatment A, the outcome T, and the confounders X, and given a time horizon h and using the do-calculus notation, we define the average treatment effect (ATE) of antibiotic-loaded bone cement usage on prosthetic survival as ψh = P[T > h|do(A = 1)] − P[T > h|do(A = 0)]. Under identifiability conditions, ψh can be expressed in terms of the observed variables, dropping the do-operator, as ψh = P[T > h|A = 1, X] − P[T > h|A = 0, X]. Nevertheless, note that in survival analysis we do not always get to measure Ti for each unit in our data sample, as some units might be censored. Thus, for our sample we define the censoring time Ci as the time at which the i-th unit gets censored, together with ∆i, a censoring indicator such that ∆i = 1{Ti > Ci}, and Ui = Ti ∧ Ci (with ∧ the logical and operator). The goal, then, is to estimate ψh using Ui and ∆i instead of Ti. Similarly, recall that the secondary goal of the study was to analyze the effect of antibiotic-loaded bone cement on prosthetic survival in specific subgroups of the population, defined by the confounders. Thus, we define the conditional average treatment effect (CATE) for the j-th confounder, as πh(xj ) = P[T > h|do(A = 1), Xj = xj ] − P[T > h|do(A = 0), Xj = xj ], and then follow an analogous logic as with the ATE. The provided definitions of the ATE and the CATE imply that they represent the difference in survival probability between the antibiotic-loaded bone cement group and the plain cement group (in the case of the ATE, in the whole population; in the case of the CATE, in a particular subgroup defined by the values of a given confounder). The sign of those quantities will indicate whether the treatment with antibiotic-loaded bone cement increases (when positive sign) or decreases (when negative sign) the survival probability of the prosthesis, and the value will indicate the size of such effect.

For a detailed explanation on the implementation of the CATE and ATE estimators with causal survival forests, see Yifan Cui et al. [1].

**Descriptive table of confounder variables stratified by ALBC or plain cement**

The confounder variables included for analysis were patient’s age, as a continuous variable in years; sex assigned at birth, as a dichotomous variable (woman or man); obesity, diabetes, rheumatoid arthritis and alcohol abuse, as dichotomous variables (yes or no); smoking status, as a categorical variable (smoker, non-smoker, former smoker; missing values treated as non-smoker); body mass index (BMI) as a continuous variable in kg/m2 (missing values imputed with the average stratified by age group); Charlson comorbidity index and Elixhauser index, as continuous variables; hospital category, as a categorical ordinal variable with five categories (between 1 and 5); primary surgery year, as a categorical ordinal variable (from 2011 to 2020); surgery duration, as a continuous variable in minutes (missing values assigned with the average stratified by hospital. Note that the hospital category classifies hospitals regarding their size and specialization level, and it is a categorization established by CatSalut. Category 1 is for high technology, reference hospitals, while Category 5 is for regional, basic hospitals. Alcohol abuse was defined as in the definition of the Elixhauser index [26].

Supplementary table 1 presents a descriptive analysis of the most relevant confounders, stratified by the treatment variable. Plain cement was used in 9,656 (42.4%) cases and ALBC in 13,125 (57.6%) cases. Small although statistically significant differences were found for sex and ALBC, and for age and ALBC. No significant differences were found regarding the analyzed comorbidities and ALBC, although the small differences observed in the Charlson and Elixhauser indexes were statistically significant. Finally, some larger differences were observed in the variables of smoking status, surgery year and hospital category. The most relevant one is the steady increase of ALBC usage along the years, going from a 46.74% in 2011 to an 84.16% in 2020 (of all surgeries in each year, respectively).

**Unstable values of the ATE**

Supplementary figure 1, top, shows values of the ATE with respect to horizon time. We consider that the drop that is observed after 130 months is not a real effect but a result of the numerical instabilities of the employed method when the time horizon is too large for the characteristics of our dataset. For proving this, see Supplementary figure 1, bottom, where we have depicted the number of unstable estimates for each time horizon value. An unstable estimate is defined as a data point in the dataset where the censoring probability, the treatment propensity or both are bigger than 0.95 or smaller than 0.05. We can see in the figure that after around 130 months the number of unstable points increases noticeably.

**CATE values across the whole horizon-confounder ranges**

Supplementary figures 2 to 15 present the CATE for the different confounders. For continuous confounders, the CATE is represented as a contour map, with values coded by colors in a color bar. The x-axis contains the time horizon and the y-axis the confounder values. For categorical confounders, we depict as many lines as categories, the x-axis containing the time horizon and the y axis the CATE value. Recall the notes about interpretation of the CATEs: 1) all CATEs represent the increase (if positive) or decrease (if negative) of the survival probability of prostheses, as a consequence of the usage of antibiotic-loaded bone cement, for given time horizons and for given subpopulations based on confounders; and 2) all CATEs are expressed as fractions of 1.

**Relevance of CI methods**

The assumptions and methodological choices of causal inference have an impact in the obtained results. This highlights the importance of making sound and transparent choices for assumptions and methods. We conducted an experiment modifying the DAG as per the premise that in Catalonia the choice of ALBC or plain cement is based solely on the hospital where the surgery was performed. This would be the case if, for instance, each hospital had a policy for the usage of antibiotic-loaded bone cement or plain cement depending only on its category and not on any other confounder. The DAG in Supplementary figure 17 reflects such scenario.

As it can be seen, all arrows to the treatment have been erased, leaving only the one coming from hospital category. Under this DAG, in order to obtain an unbiased estimate of the effect of antibiotic-loaded bone cement on prosthetic survival, it is necessary that we control not only for hospital category, which is the only variable directly affecting the treatment, but for the surgery year, the surgery duration, and the cement viscosity. This is due to the existing relationships among those variables, which create biasing paths between them, the treatment, and the outcome. Without the usage of a DAG, it would be much less intuitive to identify the need to control for those variables.

Supplementary figure 18 shows the ATEs under both DAGs, the one in Figure 2 of the main text and the one in Supplementary figure 17. Both ATEs differ. This fact highlights even further the importance of using DAGs for representing causal problems, as their structure has a direct impact in the magnitude of the estimated effects.

References

1. Cui Y, Kosorok M, Sverdrup E, Wager S, Zhu R (2023) Estimating heterogeneous treatment effects with right-censored data via causal survival forests, Journal of the Royal Statistical Society Series B: Statistical Methodology, 85(2):179–211, <https://doi.org/10.1093/jrsssb/qkac001>
